# Emergent solution based IGZO memristor towards neuromorphic applications†

**DOI:** 10.1039/d1tc05465a

**Published:** 2022-01-10

**Authors:** Raquel Azevedo Martins, Emanuel Carlos, Jonas Deuermeier, Maria Elias Pereira, Rodrigo Martins, Elvira Fortunato, Asal Kiazadeh

**Affiliations:** CENIMAT/i3N Departamento de Ciência dos Materiais, Faculdade de Ciências e Tecnologia (FCT), Universidade NOVA de Lisboa (UNL), and CEMOP/UNINOVA 2829-516 Caparica Portugal a.kiazadeh@fct.unl.pt e.carlos@campus.fct.unl.pt

## Abstract

Solution-based memristors are emergent devices, due to their potential in electrical performance for neuromorphic computing combined with simple and cheap fabrication processes. However, to achieve practical application in crossbar design tens to hundreds of uniform memristors are required. Regarding this, the production step optimization should be considered as the main objective to achieve high performance devices. In this work, solution-based indium gallium zinc oxide (IGZO) memristor devices are produced using a combustion synthesis process. The performance of the device is optimized by using different annealing temperatures and active layer thicknesses to reach a higher reproducibility and stability. All IGZO memristors show a low operating voltage, good endurance, and retention up to 10^5^ s under air conditions. The optimized devices can be programmed in a multi-level cell operation mode, with 8 different resistive states. Also, preliminary results reveal synaptic behavior by replicating the plasticity of a synaptic junction through potentiation and depression; this is a significant step towards low-cost processes and large-scale compatibility of neuromorphic computing systems.

## Abstract 2

Solution-based memristors are emergent devices, due to their potential in electrical performance for neuromorphic computing combined with simple and cheap fabrication processes.

## Graphical abstract

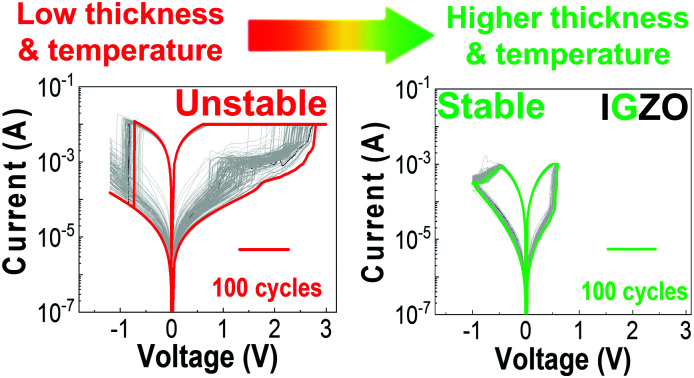

## 1. Introduction

Over the years, there has been an increasing demand to build devices with increased speed, larger capacity for storage and lower power consumption due to the emergent requirements in Internet of Things (IoT) and the artificial intelligence (AI) era. Non-volatile memory devices (NVMs) are one of these ubiquitous devices and are typically known for their ability to store information for long periods of time (over 10 years).^1^ Currently, flash memory devices are the most common NVM, presenting a high endurance and speed performance.^2,3^ However, this technology is reaching its physical limitations and will be replaced by other opportune NVM in the future. Memristors are emergent NVMs that are highly promising and a viable alternative to flash memory devices.^4,5^ These devices possess a faster operation, good endurance, multilevel cell operation and scalability.^6^ Regarding their electrical performance, memristors are based on a reversible resistive switching (RS) mechanism between a low resistance state (LRS) and a high resistance state (HRS). These state changes are due to redox reactions in valence change mechanism (VCM) memristors, that create and break conductive filaments (CFs), when a voltage stimulus is applied to the device. A CF is typically formed by the migration of oxygen anion species or by the electromigration of metal cations in the active layer.^7^

Neuromorphic computing has been a modern way to overcome the problems faced in Von Neumann's architecture. This approach emulates the function of a human brain with extremely low power consumption.^8^ In the biological process, synapses perform learning and memory functions by modulating the connection between neurons.^9^ Thus, one of the main interests in memristors is for artificial neural networks (ANNs), as a synaptic device.^10,11^ The resistance of the memristors can operate as synaptic weight, which can be updated by changing the resistance states of the memristor device. However, in the deep neural network (DNN) approach, the memristors only require a linear and symmetric response to the plasticity characteristic, whereas in real synapses time and frequency must be considered.^12^

In order to be compatible with roll-to-roll large-scale manufacturing and flexible production techniques,^13^ amorphous metal oxide semiconductor (AOS) materials appeared as an alternative to conventional Si technology, especially amorphous indium gallium zinc oxide (IGZO).^14^

IGZO has been widely studied and applied in vacuum processed and solution-processed thin-film transistors (TFTs), due to its high transparency, carrier mobility, and environmental stability.^15–17^ Building AOS–memristor devices by adopting techniques of mature flexible semiconductor electronic technology is vital for low-cost flexible ANN systems, since both electronic devices (memristors and TFTs) can share the same material, processing steps and technology.

In this respect, IGZO exhibits RS characteristics, which are ideal for the active layer of a memristor; however, there are few reports on solution processing memristor devices.^18,19^ The RS mechanism strongly depends on oxygen vacancies (*V*_o_) present in IGZO, which through solution processes can be controlled by changing gallium concentration. A change in IGZO molar proportion, particularly an increase of gallium, will suppress the generation of *V*_o_, responsible for carrier transportation.^18^ One promising method is solution combustion synthesis (SCS), used to convert precursor solutions into oxides with the addition of a fuel that leads to the initiation of a combustion reaction. The main advantage of this method is the reduction of the thermal budget required to initialize precursor conversion.^20^ This allows the fabrication of memristor devices *via* coating and printing techniques and the possibility of flexible substrates as long as a compatible annealing temperature is used.^21–23^ It is noteworthy that these processes are scalable, much simpler and cheaper than the commonly used vacuum techniques.^24,25^

In general, solution-processed memristor devices are novel, and the technology is not yet as mature as the vacuum processed ones. High quality films are extremely dependent on the fabrication methods (furnace/oven, hotplate annealing, ultraviolet photochemical activation, and microwave irradiation) and conditions which are still under optimization.^6^ Cho *et al.* showed that microwave irradiation can provide high yield and uniform IGZO memristors in a controlled environment (nitrogen).^26^ However, the main limitation of using this technique is the scale-up to roll-to-roll (R2R) compatible techniques of solution-based memristors. Table 1 summarizes the state of the art of solution-based IGZO memristor devices.

**Table 1:** Performance comparison of solution-based IGZO memristors

Year	BE^a^/TE^b^	*T*_max_ (°C)	*R*_ON/OFF_/MLC	Retention (s)	Endurance (cycles)	Neuromorphic applications
2012^18^	Al/Al	370	2.7/No	n.d.	10^2^	No
2014^27^	Pt/Ti	300	3 × 10^1^/No	10^4^	9 × 10^1^	No
2014^19^	Pt/Pt	130	>10/No	10^4^	10^2^	No
2017^28^	Ti/Ti	350	>10/No	10^4^	10^2^	No
2018^29^	ITO/Pt	150	Approx. 10^3^/No	10^4^	10^4^	No
2020^30^	Ni/Pt	250	Approx. 10^2^/No	10^4^	2.5 × 10^2^	No
2021^31^	Al/ITO	350	n.a./Yes	n.a.	n.a.	Yes
2021^26^	Pt/Ti	MA^c^	10^1^/Yes	10^4^	10^3^	Yes
This Work	Ti/Pt	200	10^2^/Yes	10^5^	10^2^	No
	Ti/Au	300	10^2^/Yes	10^5^	10^2^	Yes

a Bottom electrode.

b Top electrode.

c Microwave annealing, n.a. – not available.

In this work, we report on solution-based IGZO memristors and the study of the influence of annealing temperature and active layer thickness on the device performance in an air environment. The optimized IGZO (1 : 3 : 1) memristors show bipolar resistive switching behavior with low variability, good endurance, and a retention time of up to 10^5^ s in ambient air, which surpasses the current state of the art. These devices reveal a multilevel cell (MLC) characteristic, achieving 8 different resistive states, 3 bits per cell. Some preliminary results on the synaptic behavior of the solution-based IGZO memristors through potentiation and depression are also shown, proving that these devices are suitable for neuromorphic applications.

## 2. Experimental section

### 2.1. Precursor solution synthesis and characterization

Indium(iii) nitrate hydrate (In(NO_3_)_3_·*x*H_2_O, Sigma-Aldrich, 99.9%), gallium(iii) nitrate hydrate (Ga(NO_3_)_3_·*x*H_2_O, Sigma-Aldrich, 99.9%) and zinc nitrate hexahydrate (Zn(NO_3_)_2_·6H2O, Sigma-Aldrich, 98%) were separately dissolved in 2-methoxyethanol (2-ME, C_3_H_8_O_2_, Fisher Chemical, 99%) to produce metal precursor solutions with a concentration of 0.2 M. The fuel (urea, Sigma, 99%) was added to each solution and maintained under constant stirring for 1 h. In order to guarantee the redox stoichiometry of the reactions, the urea to indium nitrate, gallium nitrate and zinc nitrate molar proportions were (5/2):1, (5/2):1 and (5/3):1, respectively. IGZO precursor solution was prepared by combining the three precursor solutions made, to obtain an In_2_O_3_ : Ga_2_O_3_ : ZnO molar ratio of 1 : 3 : 1 with a 0.2 M concentration. The precursor solution was stirred for at least 36 h at room temperature and filtrated using a PTFE filter (0.45 μm) before use.

Thermogravimetry and differential scanning calorimetry (TG-DSC) (Netzsch, TG-DSC-STA 449 F3 Jupiter) were performed for the IGZO (1 : 3 : 1) solution under an air atmosphere up to 550 °C at a 10 °C min^−1^ heating rate in an aluminum crucible.

### 2.2. Thin film deposition and device fabrication

MIM structures were fabricated on corning glass substrates (Soda-lime glass). Prior to deposition, all substrates were cleaned as mentioned in a previous report.^22^ The bottom electrode, a Ti/Pt bilayer of 30 and 30 nm, was first deposited on the substrate by e-beam evaporation (homemade apparatus). Then, the IGZO thin films were deposited by spin coating for 35s at 2000 rpm (Laurell Technologies), forming a single layer. Each deposition was followed by an immediate hotplate annealing at 200 °C or 300 °C for 30 min in an air environment (relative humidity (RH): 43–63% at room temperature). This process was repeated several times (1, 3, 5 and 7 layers), with a 10 min UV/Ozone surface treatment between each deposition. After thin film fabrication, a multilayer of Ti/Au, 6 nm and 60 nm, respectively, was deposited by e-beam evaporation as the top electrode (25 devices) using a physical mask for electrode (area of 1.96 × 10^−3^ cm^2^) patterning.

### 2.3. Thin film and device characterization

Optical properties were obtained using a PerkinElmer lambda 950 UV/VIS/NIR spectrophotometer by measuring transmittance (*T*) in the wavelength range of 250–2500 nm. The optical characterization can be found in the ESI† (Fig. S1). Fourier transform infrared (FTIR) spectroscopy data of thin films deposited on Si substrates were recorded using an attenuated total reflectance (ATR) sampling accessory (Smart iTR) equipped with a single bounce diamond crystal on a Thermo Nicolet 6700 Spectrometer. The spectra were acquired as reported in the ESI† (Fig. S2). Atomic force microscopy (AFM, Asylum MFP3D) was performed to analyze the topology of the optimal IGZO sample (Fig. S3 in the ESI†).

X-Ray photoelectron spectroscopy (XPS) was measured using a Kratos Axis Supra spectrometer. A monochromatic Al K α source was used with an aperture of 110 μm and the analyzer was set to pass an energy of 80 eV. For depth profiling, an argon cluster of 500 atoms was used, with a kinetic energy of 10 keV, and scanned over 1.5 mm^2^. The data were analyzed with CasaXPS software.

Spectroscopic ellipsometry was used to measure the thin film thickness, with an energy range from 1.5 to 5.5 eV and an incident angle of 45° using the Yvon Uvisel system. The acquired data were modulated using the DELTAPSI software and the fitting procedure was done pursuing the minimization of error function (*χ*2).

The quasi-static current–voltage (*I*–*V*) characteristics and the pulse studies of the devices were measured using a Keithley 4200 SCS semiconductor analyzer connected to the Janis ST-500 probe station. The bias was applied to the top electrode while maintaining the bottom electrode connected to the ground. The speed of the measurements was at normal mode and the integration time was in auto setting.

## 3. Results and discussion

**Fig. 1:**
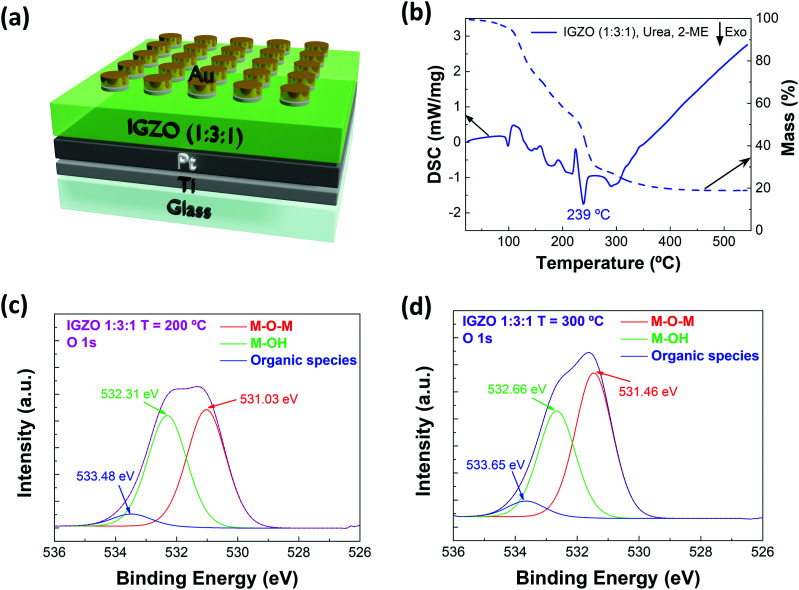
Schematic (a) of the IGZO (1 : 3 : 1) memristor device structure; (b) TG-DSC curves of IGZO (0.2 M) precursor solution with a molar ratio of 1 : 3 : 1; and XPS surface spectra of samples annealed at (c) 200 °C and (d) 300 °C.

Fig. 1(a) illustrates a sample with the produced memristors on a glass substrate. The bottom and top electrodes are Ti/Pt and Ti/Au, respectively, and IGZO is the active layer.

To highly improve the performance of the memristor, the right molar proportion and doping on the IGZO layer need to be well defined.^6^ To obtain high quality solution-based IGZO memristors, the study of gallium concentration is fundamental to improve the RS behavior since it suppresses *V*_o_ generation, and therefore reduces the mobility.^18^

TG-DSC was performed on the IGZO solution with a molar ratio of 1 : 3 : 1, as shown in Fig. 1(b). The TG data points confirm that precursor conversion is near complete below 250 °C, with few reactions at higher temperatures related to some residual organics. The DSC data points reveal one main exothermic peak during the combustion reaction at 239 °C with a mass loss of approximately 34%.

The optical transmittance of the IGZO thin films was measured to evaluate their transparency. The study shows a decrease from 90% to 87% in transmittance with the increase of deposited layers under all conditions, as depicted in Fig. S1 in the ESI.† The produced IGZO thin films were also characterized with ATR-FTIR in order to analyze the chemical bonds present, as shown in Fig. S2 in the ESI.† AFM analysis (Fig. S3 in the ESI†) shows that a uniform and smooth surface was obtained. The IGZO thin films under optimal conditions present a surface roughness lower than 0.3 nm.

XPS analysis was performed in two samples annealed at different temperatures. Fig. 1(c and d) present the XPS surface spectra of the samples annealed at 200 °C and 300 °C, respectively. In both cases, O 1s is deconvoluted into three main peaks, each one corresponding to a different type of bond present in the sample. The first peak (red) is related to M–O–M bonds, the second peak (green) is related to M–OH bonds and the third peak (blue) is due to the water and organic species adsorbed on the surface. These results reveal that at 300 °C more metal–oxygen bonds and less oxygen vacancies are present compared with the peak intensity at 200 °C, which indicates that at higher temperature the devices are more resistive. The interface between the IGZO and the Pt bottom electrode was studied by argon cluster depth profiling; the respective spectra are presented in Fig. S4 and S5 in the ESI.† After most of the IGZO layer was removed, a significant C 1s emission could be detected in the sample annealed at 200 °C (after 1100 s etch time), whereas the C 1s signal remained below the detection limit in the sample annealed at 300 °C (after 1000 s etch time). This indicates that organic compounds remain inside the film after annealing at 200 °C.

Spectroscopy ellipsometry was performed to measure the thickness of the active layers as depicted in Fig. S6 in the ESI.† An increase in deposited IGZO layers leads to an increase in thickness, regardless of the temperature used during the production of the memristors.

**Fig. 2:**
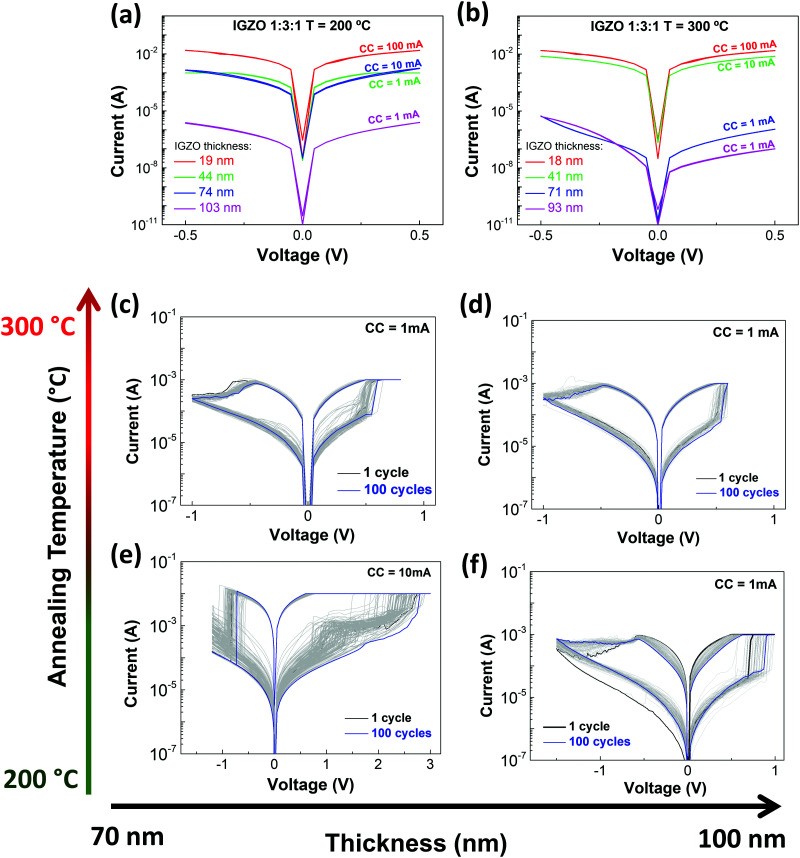
Ti/Pt/IGZO/Ti/Au electrical characterization. Pristine states of memristors with different IGZO thicknesses and annealed at (a) 200 °C and (b) 300 °C. *I*–*V* characteristics obtained from endurance tests during 100 cycles of memristors with different IGZO thicknesses: (c and d) devices annealed at 300 °C; (e) and (f) devices annealed at 200 °C.

Concerning electrical performance, the pristine state of the IGZO memristors is considered the first *I*–*V* characteristic obtained. Fig. 2(a and b) reveal the pristine state evolution when there is an increase of the active layer thickness. Under both temperature conditions, the resistance increases with higher thickness. Also, devices annealed at 300 °C show a similar tendency in behavior, unlike devices annealed at 200 °C, that have variable pristine curves. At 300 °C the combustion reaction is completed and so, all the nitrate compounds are reduced to their metallic states. This can be due to the fact that the IGZO combustion reaction occurs until 250 °C as observed in Fig. 1(b), indicating that the use of lower annealing temperatures leaves residues of organic compounds, affecting their performance. This is corroborated by XPS analysis, which indicates that devices annealed at 200 °C have more M–OH groups and organic compounds, confirming that the combustion reaction was not completed.

The stoichiometry used promotes a high electrical resistivity of the devices since the gallium oxide present in IGZO is an insulator material.^27^ By increasing the thickness of the active layer in the memristor, the device gets more resistive and consequently it decreases its off-state current, as observed in the pristine curves (Fig. 2(a and b)). The results achieved with devices under these conditions can be easily reproducible.

Further electrical tests were performed only on the samples with higher IGZO thickness since these showed a higher reproducibility. The conditions studied were memristors with higher IGZO thickness (*i.e.*, number of deposited layers) annealed at 200 °C or 300 °C. To activate the RS, an electroforming step was performed, as shown in Fig. S7 in the ESI.† All the devices formed under a positive bias, between 1 V and 2.5 V. A current compliance (CC) had to be maintained to prevent the breakdown of the devices. The *I*–*V* curves exhibit bipolar resistive switching under a CC of 10 mA for one device produced at 200 °C with a lower IGZO thickness and 1 mA for those under other conditions, as shown in Fig. 2(c–f). The *I*–*V* characteristics were measured with a controlled voltage through the top electrode and under consecutive DC voltage sweeps.

The memristors reveal good endurance and a uniform switching behavior, although the devices annealed at 200 °C present a higher variability (set and reset), as depicted in Fig. S8 in the ESI.† The increase in temperature and active layer thickness reduces the deviation on set and the operating voltage. For a gradual reset there is no deviation in voltage, which is the case for devices annealed at 300 °C and devices with a thicker active layer annealed at 200 °C. In terms of conductance fluctuation, presented in Fig. S9 in the ESI,† both on and off states show higher variability in devices produced at 200 °C. Memristors annealed at 300 °C have smaller changes in both states. The annealing temperature has a higher influence on the conductance fluctuation than the thickness of the active layer. Thus, electrical results are in accordance with the material characterization studies (TG-DSC and XPS). The memristors annealed at 200 °C have less metal–oxygen bonds in their structure, resulting in less stable devices. However with annealing at 300 °C, the combustion reaction to form metal–oxygen bonds is completed. The produced devices have high stability and show great reproducibility being highly desirable for further upscaling techniques and applications.

Concerning IGZO thickness, the thicker the layer the more stable the device, even the ones produced at lower temperatures. This is due to the increased number of IGZO depositions that results in an increased number of annealing steps and fills the possible defects, improving the thin-film quality. Although a higher thickness reduces the probability of leakage, the annealing temperature is crucial to obtain a denser thin-film. Low annealing temperatures leave more organic residues than desirable, which interferes with the CF formation and consequently with the electrical performance.

Nevertheless, the conditions that show the best endurance and stability are those of the device depicted in Fig. 2(d) annealed at higher temperature. Fig. S10 in the ESI† compares the set voltage of devices from two different batches that have the optimal thickness for a good electrical performance. Both batches present some variability due to the non-patterned device structure.

Fig. S11 in the ESI† represents a typical retention of the memristors in ambient air. This test reveals a good retention of up to 10^5^ s in air, with an *R*_ON/OFF_ ratio of 10^2^ and no significant degradation of HRS and LRS, indicating that these memristors are stable and non-volatile.

**Fig. 3:**
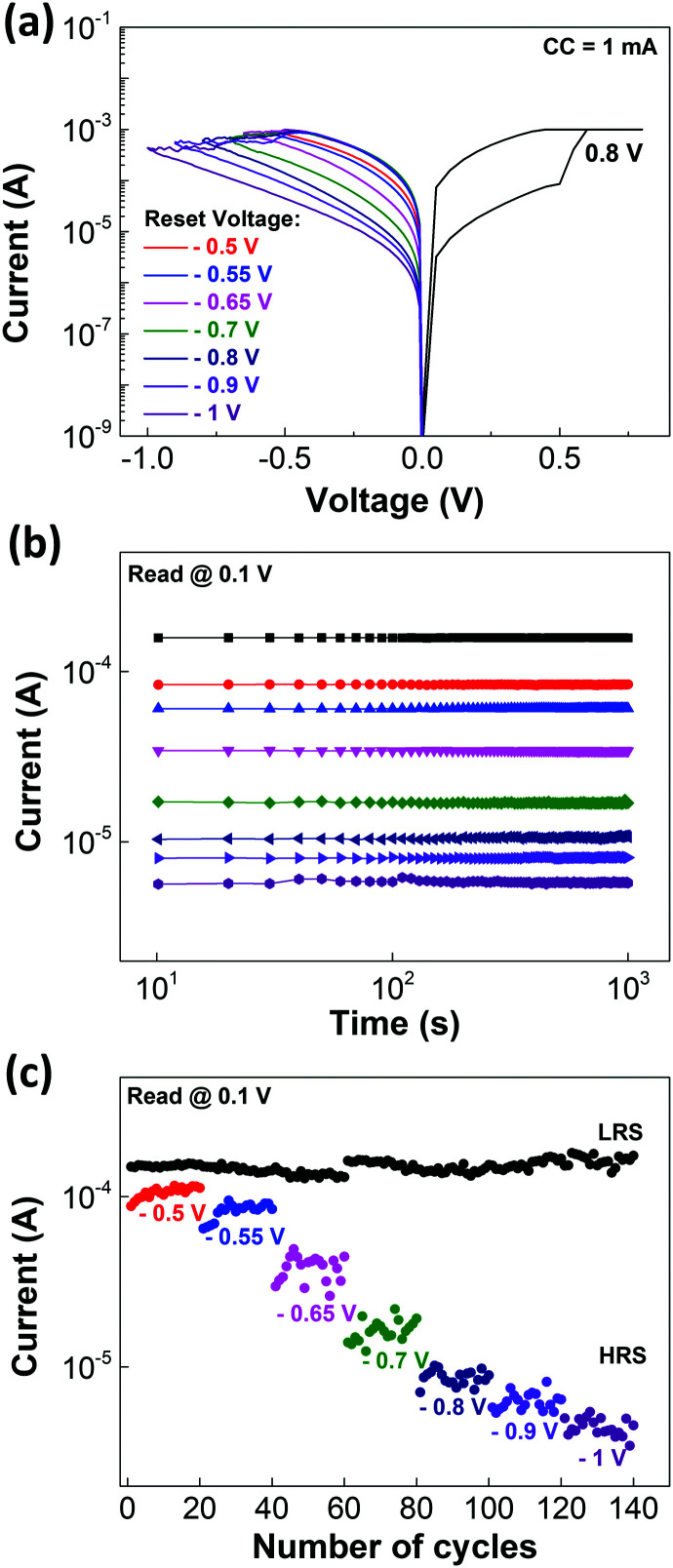
Solution-based IGZO (1 : 3 : 1) memristors: (a) reset and set *I*–*V* curves of MLC retention; (b) MLC retention characteristics with read at 0.1 V during 10^3^ s for seven different reset voltages; and (c) cycle to cycle MLC retention of the states presented in (b).

Another interesting feature of these memristors is the multilevel cell (MLC) behavior. The possibility of achieving more than two resistive states, allows the storage of more than 1 bit per cell which is highly important to achieve high densification.^32^Fig. 3(a) shows the IV curves of the MLC characteristic obtained using the reset stop voltage method^33^ to program different states. For each level programmed in the device a retention of 10^3^ s was performed as depicted in Fig. 3(b). By using this methodology, it was possible to achieve 8 different resistance states (3 bits per cell) with a cycle-to cycle variation along for each state as shown in Fig. 3(c).

Since these devices are capable of MLC storage, a feature required for neuromorphic applications,^34^ pulse measurements were performed only under the best device conditions mentioned previously.

**Fig. 4:**
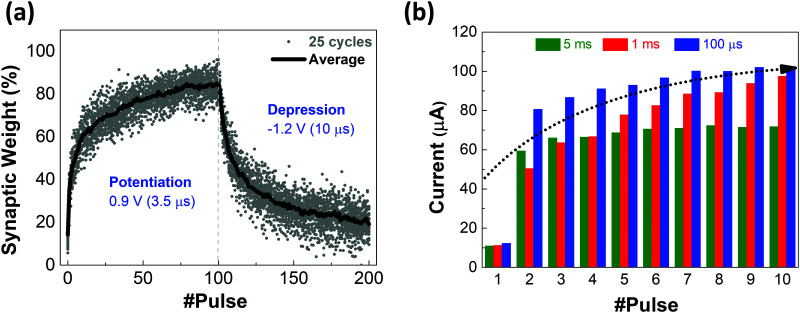
Solution-based IGZO device: (a) synaptic weight change in percentage of 25 cycles for 100 consecutive pulses (potentiation and depression) under 0.1 V voltage read. (b) Mean change of Δ*I* of current during cycles of 10 pulses with different intervals: 5 ms, 1 ms and 100 μs.

The accumulative behavior of the memristor is a key performance for in-memory computation and realized as the weight update in the training process of a neural network hardware. Preliminary results show that the device can replicate the plasticity (potentiation/depression) of a synapse, when positive or negative consecutive pulses are applied.^9^ Under a scheme of identical pulses, the current alteration can be translated to a change of synaptic weights. Fig. 4(a) shows the typical conductance response to 100 positive consecutive pulses (potentiation) immediately followed by 100 negative pulses (depression), under a 0.1 V voltage read for 25 cycles. Regarding potentiation, pulses with 0.9 V of amplitude and 3.5 μs of width show a gradual exponential growth of conductance change with a faster weight change at the beginning. Whereas, in depression, by applying pulses with −1.2 V of amplitude and 10 μs width a gradual exponential reduction of conductance is observed.

Generally, depression conductance variations to fully reset state can be better gradually controlled (also as shown in Fig. S12 in the ESI†).

Spike rate dependent plasticity as shown in Fig. 4(b) is one of the basic characteristics to demonstrate the effect of pulse rate on the synaptic weight of the device. It is one of the requirements of spiking neural network (SNN) systems where it incorporates the concept of time into operation similar to what happens in biology. Nevertheless, coding methods and efficient learning algorithms of SNN have not been widely explored yet; hence there exist many challenges for full adoption of memristor technology. On the other hand, memristors can be programmed into various resistance states by applying different pulses and are capable of in-memory computation by accumulative behavior (progressively reducing/increasing resistance) which is fundamental in training artificial neural networks (ANNs). However, the symmetry and linearity of memristor responses greatly impact the network accuracy.^35^

As a final note, for a successful design of the all-solution processed neuromorphic system, the current IGZO memristor is required to be integrated with thin film transistors as reported by our group.^24^ Such a configuration of 1T1R allows an easy and efficient programming of devices into linear and symmetric synaptic responses by modulation of the transistor channel resistance *via* gate voltage bias.

## 4. Conclusions

Solution-based memristors have a lot of potential due to their scalable production at low cost, using more environmentally friendly techniques. Yet, there are some challenges to overcome in production, such as low uniformity over a large area, something that directly affects the electrical performance of the devices in terms of reproducibility, stability, and variability. In this work, we surpass some of these issues by producing IGZO memristors using several layer depositions to minimize the stability problems. Also, the selection of an appropriate IGZO molar proportion (1 : 3 : 1) was important for improved RS characteristics. The right production methods and an adequate active layer thickness show an increased stability and device-to-device reproducibility. The MLC characteristics and synaptic behavior presented revealed promising results for neuromorphic computing for future large-scale manufacturing with diverse IoT applications.

## Author contributions

The sample fabrication and characterization were performed by R. A. Martins under the supervision of A. Kiazadeh and E. Carlos. The manuscript was prepared by R. A. Martins. All authors examined, commented, and have approved the final version of the manuscript.

## Conflicts of interest

The authors declare no competing financial interest.

## Supplementary Material

TC-010-D1TC05465A-s001
