# Supplementary material for: Emergent solution based IGZO memristor towards neuromorphic applications
Source: J Mater Chem C Mater. 2022 Jan 10;10(6):1991–8. doi: 10.1039/d1tc05465a (PMC9241358; doi:10.1039/d1tc05465a)
Supplement: TC-010-D1TC05465A-s001 [file TC-010-D1TC05465A-s001.pdf]

Electronic Supplementary Material (ESI) for Journal of Materials Chemistry C.
This journal is © The Royal Society of Chemistry 2022

Electronic Supplementary Information (ESI)

Emergent solution based IGZO memristor towards neuromorphic applications

Raquel Azevedo Martins, Emanuel Carlos, Jonas Deuermeier, Maria Pereira, Rodrigo Martins, Elvira Fortunato, and Asal Kiazadeh**

CENIMAT/i3N Departamento de Ciência dos Materiais, Faculdade de Ciências e Tecnologia (FCT), Universidade NOVA de Lisboa (UNL), and CEMOP/UNINOVA, 2829-516 Caparica, Portugal. *E-mail: a.kiazadeh@fct.unl.pt and e.carlos@campus.fct.unl.pt.

The supplementary information contains relevant data related to the material and electrical characterization of the memristors. Figure S1 (a) and (b) shows the transmittance of IGZO thin films with several thicknesses, respected to each annealing temperature. Figure S2 (a) and (b) depict the FTIR spectra of IGZO thin films annealed with different temperatures. Figure S3 shows an AFM image of an IGZO sample. Figure S4 and Figure S5 contains the XPS depth profiles performed in samples annealed at 200 ºC and 300 ºC, respectively. Figure S6 depicts the ellipsometry spectroscopy values of thickness of the active layer correspondent to the number of IGZO layers deposited. Figure S7 shows the electroforming of the IGZO memristors. Figure S8 indicates the set and reset voltage variability of each device studied. Figure S9 contains the conductance fluctuation of on and off state of each studied memristor. Figure S10 presents a histogram of the set voltage distribution for two different batches. Figure S11 shows the typical retention time of the IGZO memristors. Figure S12 shows the response of depression with different pulse intervals.

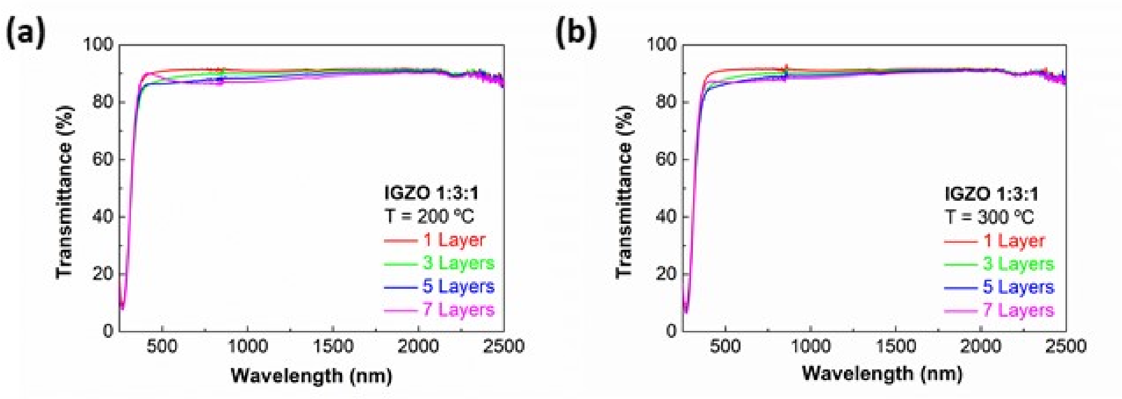

Figure S1. Optical transmittance of IGZO thin films with different thickness annealed at (a) 200 ºC and (b) 300 ºC.

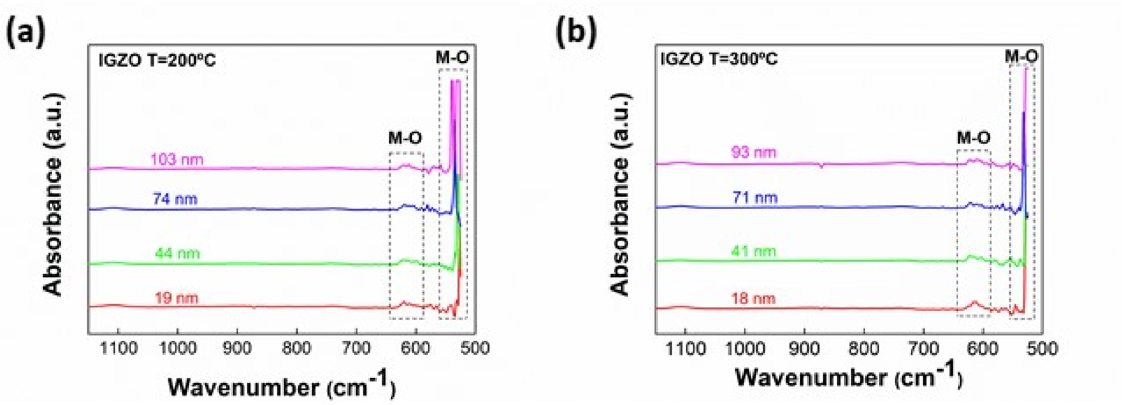

Figure S2. FTIR spectra of IGZO thin films with different thickness annealed at (a) 200 ºC and (b) 300 ºC.

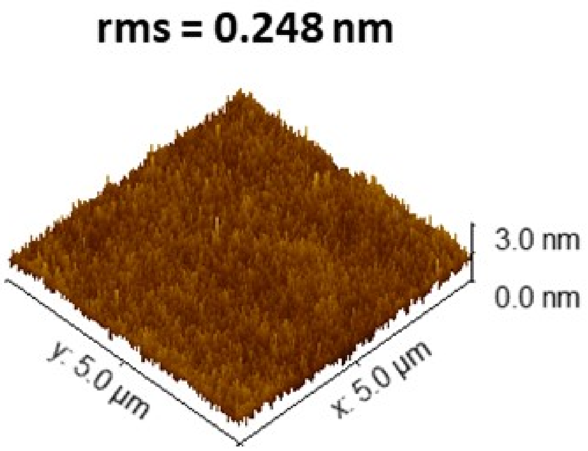

Figure S3. Three-dimensional AFM image (5 x 5 µm^2) of an IGZO thin film with ~ 90 nm thickness and annealed at 300 °C deposited in a glass/Ti/Pt substrate.

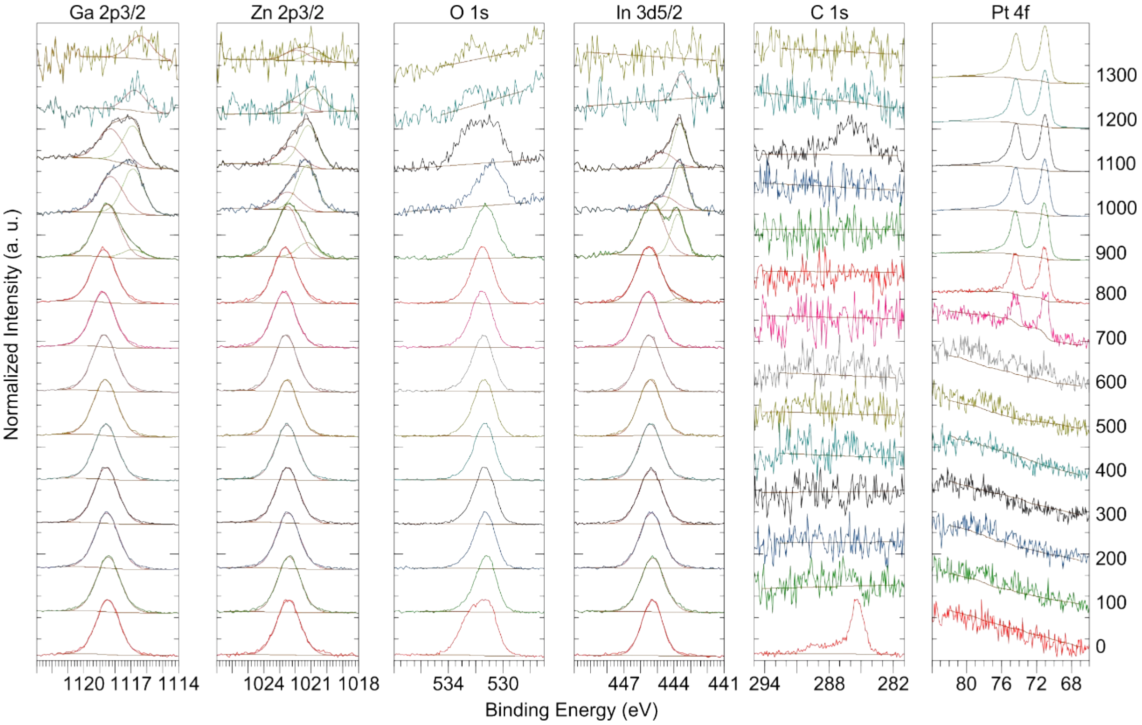

Figure S4. XPS cluster depth profile spectra of Ga 2p3/2, Zn 2p3/2, O 1s, In 5d3/2, C 1s and Pt 4f of a device annealed at 200 ºC. The etch time in seconds is displayed on the right.

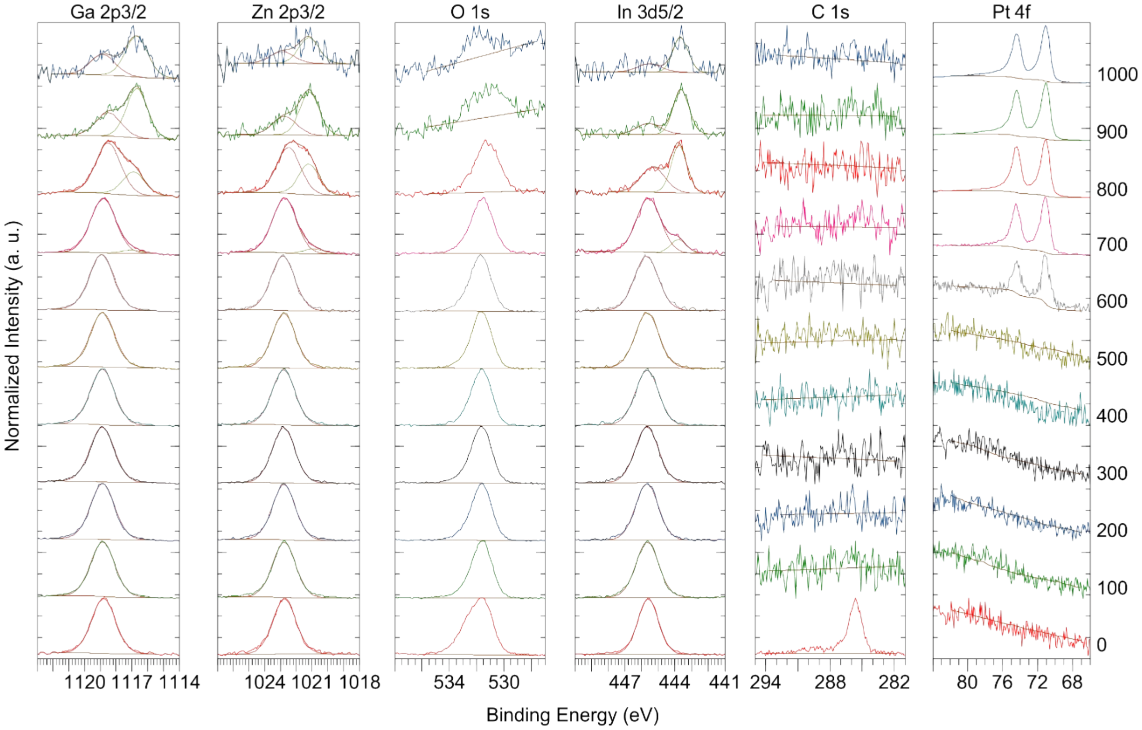

Figure S5. XPS cluster depth profile spectra of Ga 2p3/2, Zn 2p3/2, O 1s, In 5d3/2, C 1s and Pt 4f of a device annealed at 300 ºC. The etch time is displayed on the right.

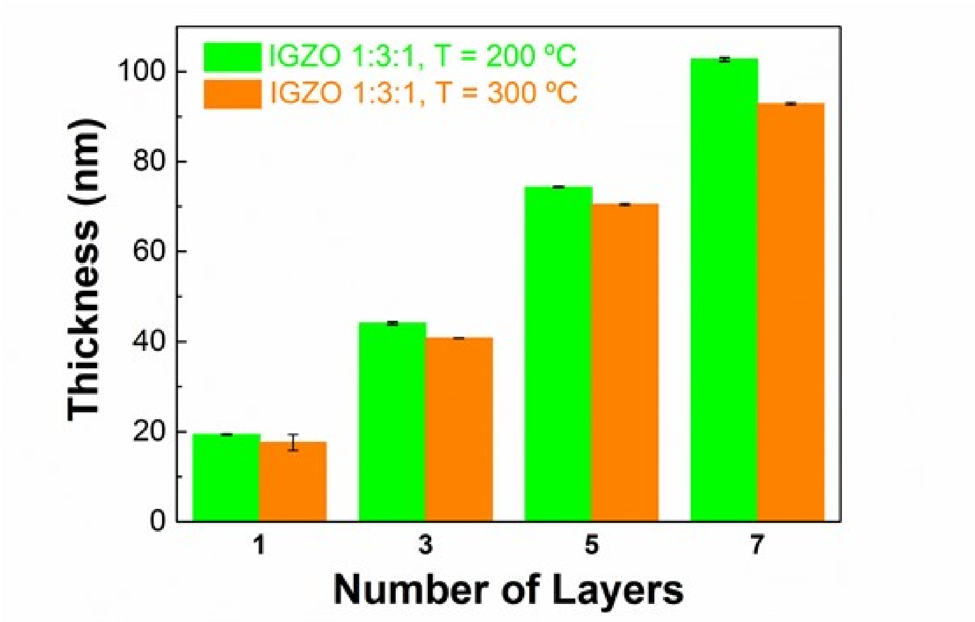

Figure S6. Spectroscopy measurements of the IGZO 1:3:1 thin films thickness (nm): annealed at 200 ºC (light green), IGZO 1:3:1 at 300 ºC (orange).

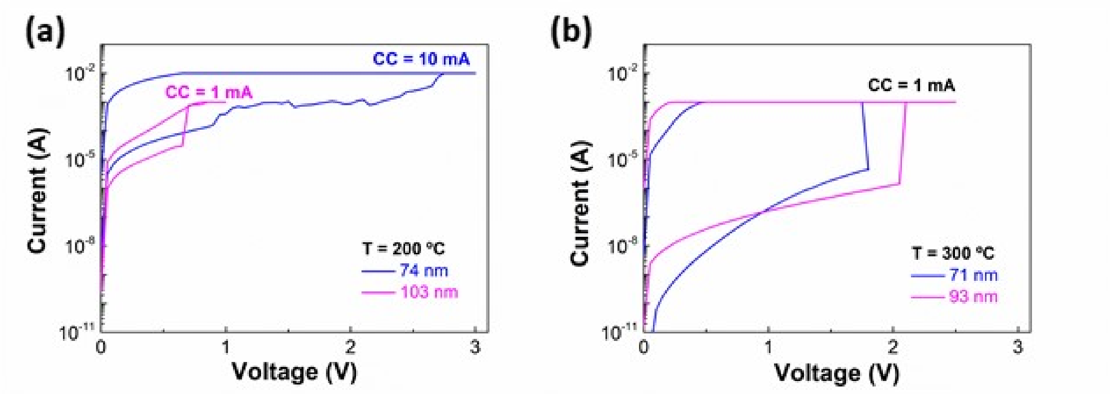

Figure S7. Electroforming I-V curves of the devices annealed at (a) 200 ºC and (b) 300 ºC.

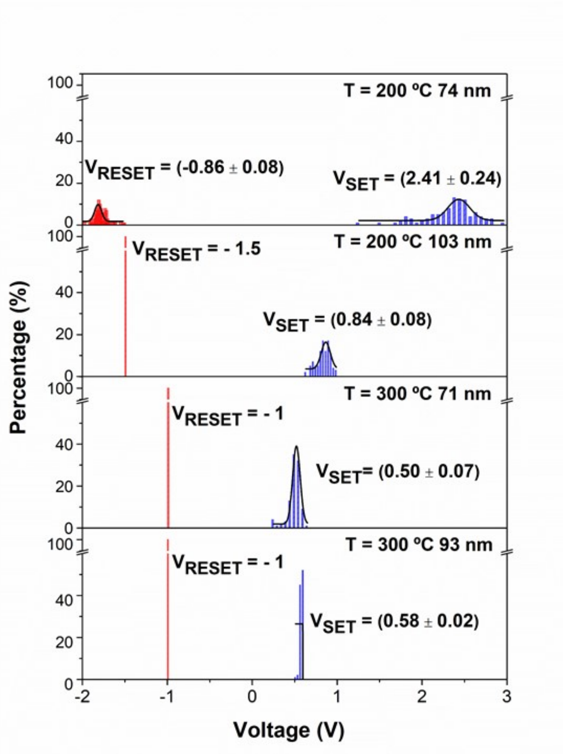

Figure S8. Set and reset average voltage of the I-V curves studied in each condition.

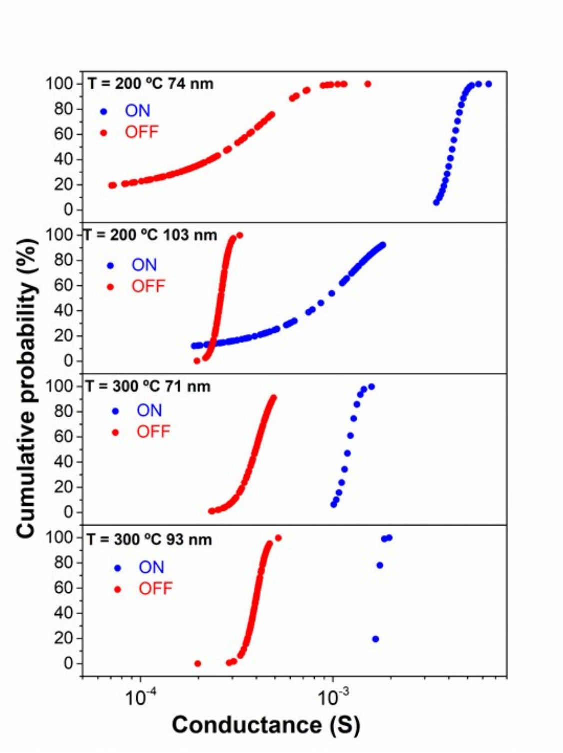

Figure S9. Conductance fluctuation of on and off states in each studied condition.

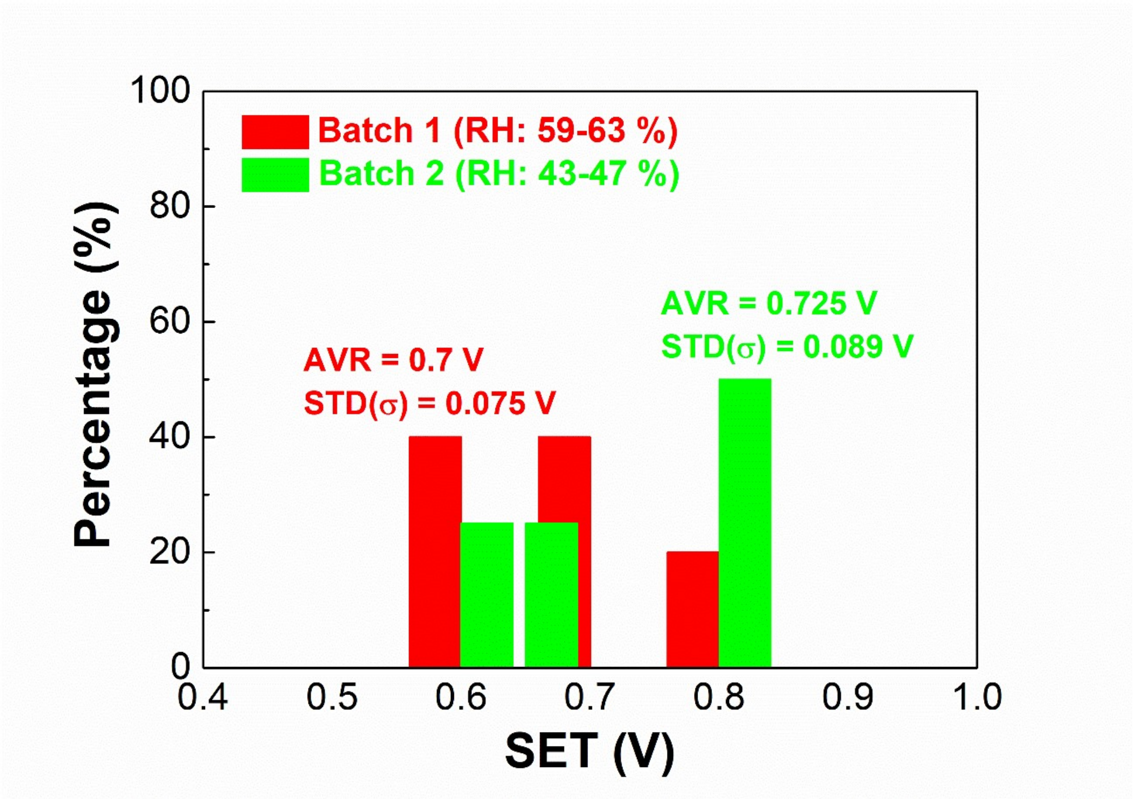

Figure S10. Histogram for the set voltage distribution for two batches under different relative humidity (RH) of the optimal memristors conditions.

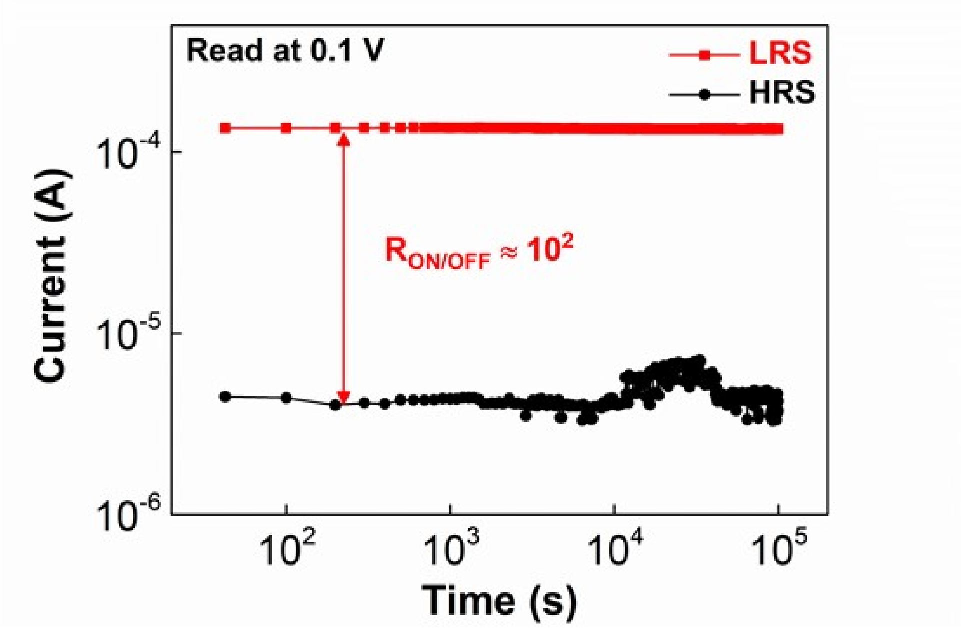

Figure S11. Typical retention time with read at 0.1 V during 10^5 s for HRS (black) and LRS (red).

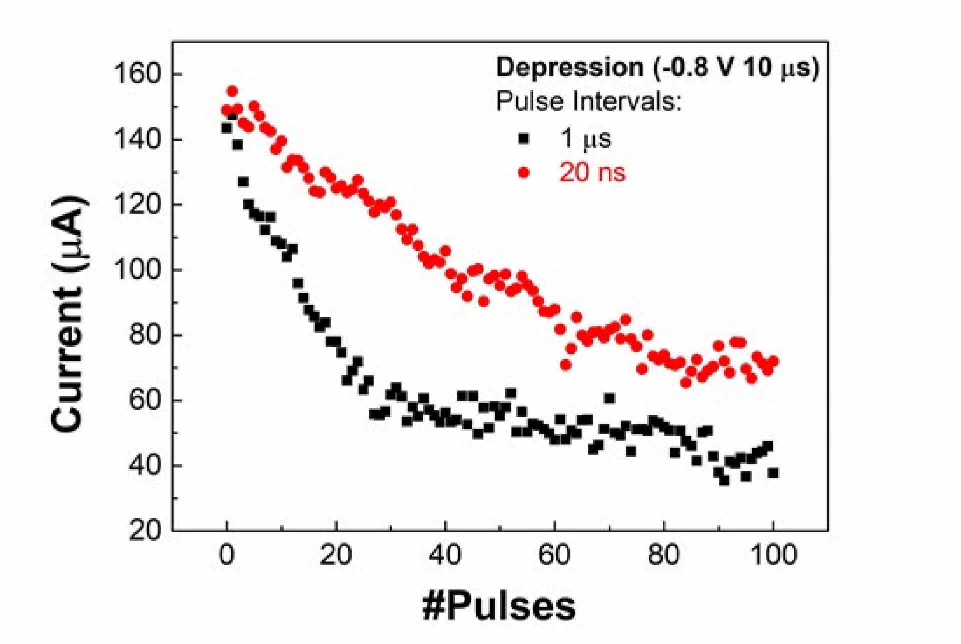

Figure S12. Conductance response, under a 0.1 V read voltage of 100 consecutive pulses with -0.8 V and 10 μs with different pulse intervals.
